# A Practical Labratory Course in Medical Chemistry

**Published:** 1883-02

**Authors:** 


**A Practical Laboratory Course in Medical Chemistry.** By JOHN C. DRAPER, M. D., LL. D., Professor of Chemistry in the Medical Department, University of New York, etc. New York: Wm. Wood & Co., 56 Lafayette Place.

This little work comprises what a young doctor ought to know about chemistry for his patient's good. Commonly there is an attempt made to teach too much chemistry to the medical student, the result being that as his time is so fully occupied with other branches which he considers more important, he neglects the study of chemistry in dispair of ever mastering it. Dr. Draper has wisely limited his course, therefore, to instructions in manipulations and to the study of tests which are useful in forming a correct diagnosis or in solving hygienic problems. The arrangement of the book is very convenient, and we think it might wisely be adopted in all medical schools where students are expected to acquire a knowledge of medicine in a two year's course.

